# Supplementary material for: The elite haplotype OsGATA8-H coordinates nitrogen uptake and productive tiller formation in rice
Source: Nat Genet. 2024 Jun 13;56(7):1516–26. doi: 10.1038/s41588-024-01795-7 (PMC11250373; doi:10.1038/s41588-024-01795-7)
Supplement: Supplementary file 1 — Supplementary Figs. 1–23. [file 41588_2024_1795_MOESM1_ESM.pdf]

nature genetics

Supplementary information https://doi.org/10.1038/s41588-024-01795-7

The elite haplotype *OsGATA8*-H coordinates nitrogen uptake and productive tiller formation in rice

In the format provided by the authors and unedited

Supplementary Figures

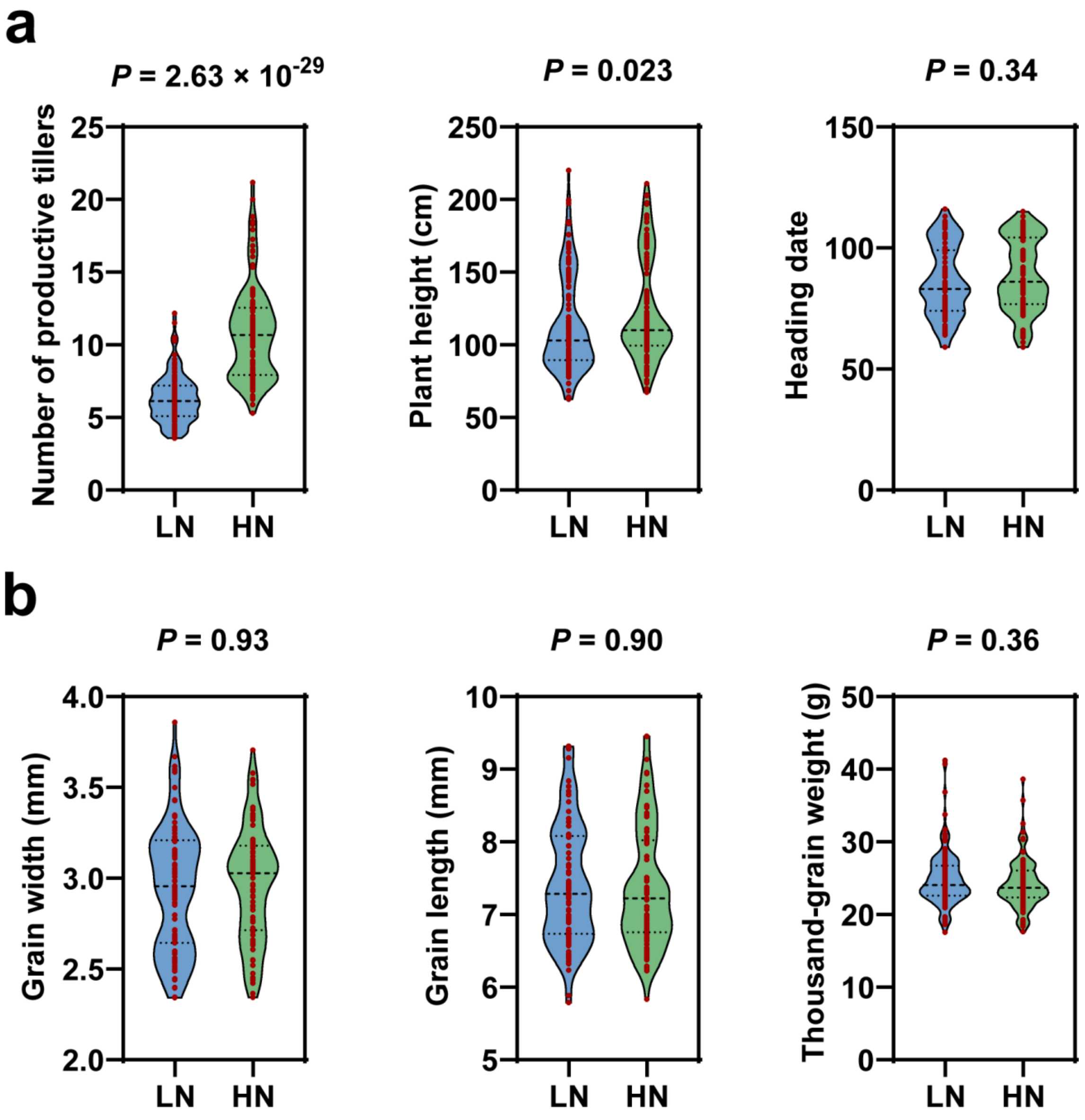

Supplementary Figure 1: The number of productive tillers and plant height positively correlate with soil nitrogen content.

(a-b) Number of productive tillers, plant height, heading date, thousand-grain weight, grain length, and grain width of 117 varieties under low and high nitrogen field conditions. Bars within the violin plots represent the 25th percentile, the median, and the 75th percentile, respectively. In **a**, **b**,

statistical significance was determined by a two-tailed Student's *t* test.

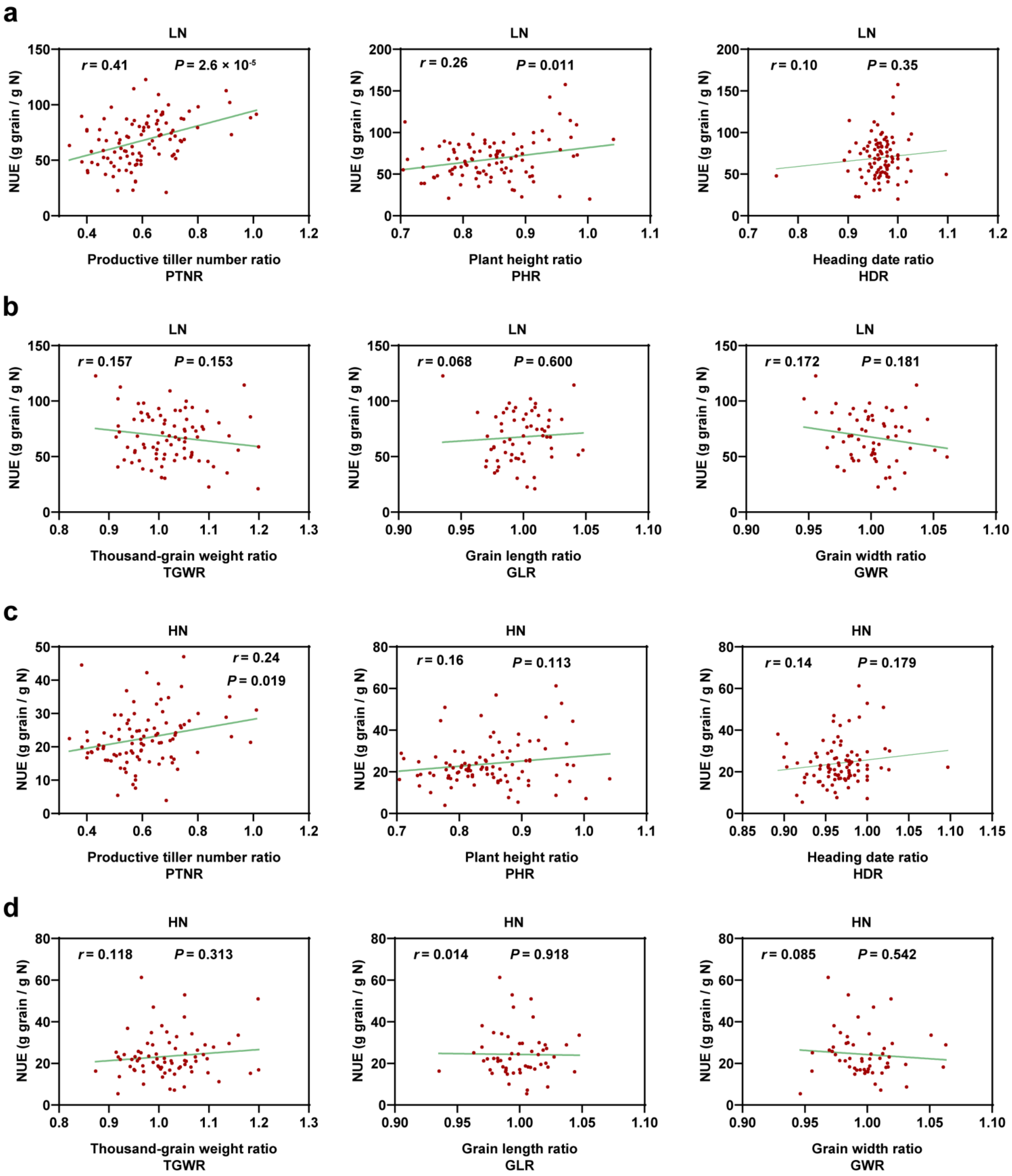

Supplementary Figure 2: PTNR and PHR positively correlate with NUE.

(a-d) Pearson's correlation coefficients were analyzed for NUE using up to 117 rice varieties with

PTNR, PHR, HDR, TGWR, GLR, and GWR under LN and HN field conditions. All *P* values are derived from two-tailed Student's *t* tests. NUE = yield per plant/average amount of nitrogen applied per plant. LN, low nitrogen (75 kg/ha net nitrogen); HN, high nitrogen (300 kg/ha net nitrogen); PTNR, productive-tiller-number ratio (productive tiller number under LN condition / productive tiller number under HN condition); PHR, plant height ratio (Plant height under LN condition / Plant height under HN condition); HDR, heading date ratio (Heading date under LN condition / Heading date under HN condition); TGWR, thousand-grain weight ratio (Thousand-grain weight under LN condition / Thousand-grain weight under HN condition); GLR, grain length ratio (Grain length under LN condition / Grain length under HN condition); GWR, grain width ratio (Grain width under LN condition / Grain width under HN condition).

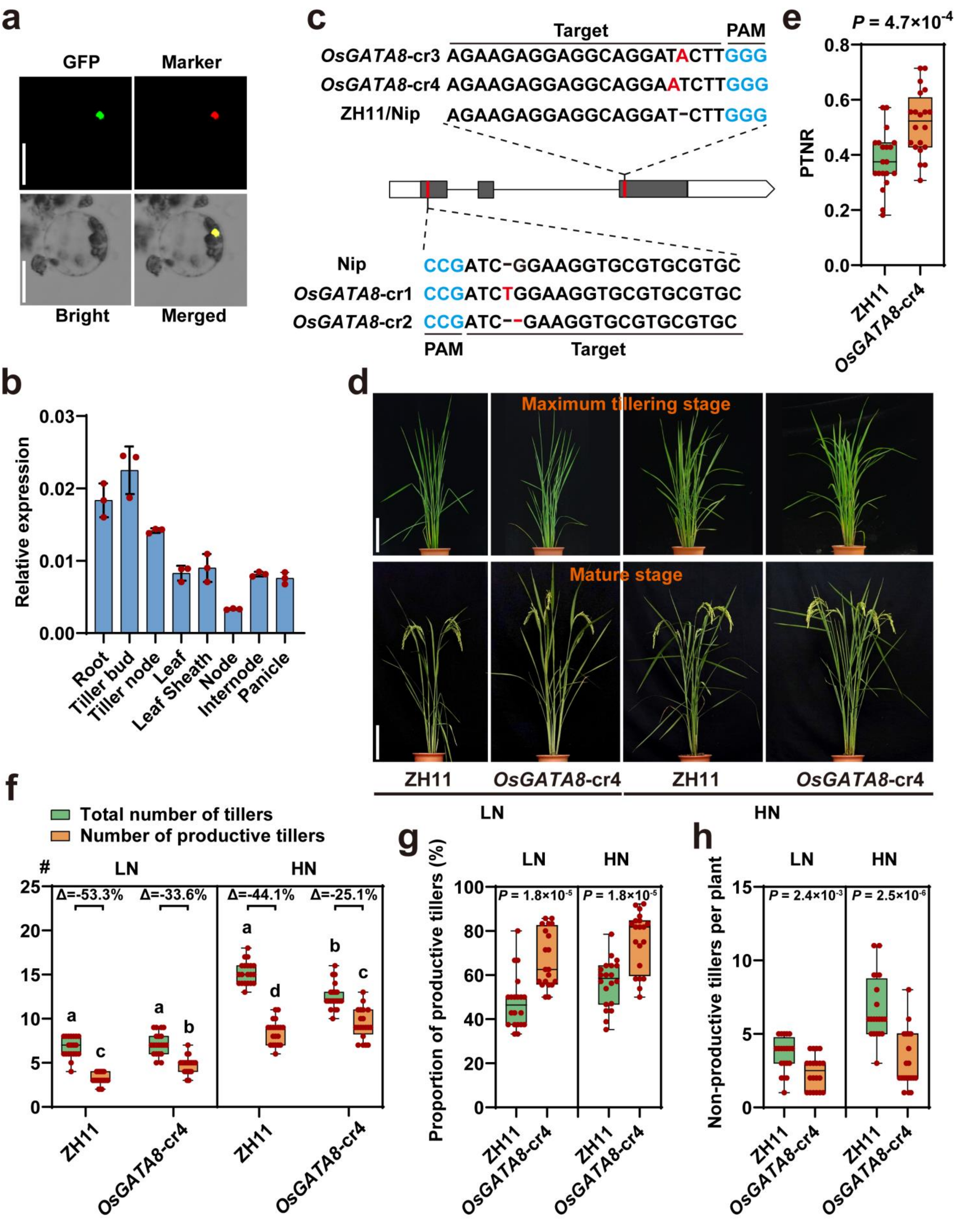
a
GFP
Marker
Bright
Merged
b
Relative expression
Root
Tiller bud
Tiller node
Leaf
Leaf Sheath
Node
Internode
Panicle
c
Target
PAM
OsGATA8-cr3 AGAAGAGGAGGCAGGATACTTGGG
OsGATA8-cr4 AGAAGAGGAGGCAGGAATCTTGGG
ZH11/Nip AGAAGAGGAGGCAGGAT-CTTGGG
Nip CCGATC-GGAAGGTGCGTGCGTGC
OsGATA8-cr1 CCGATCTGGAAGGTGCGTGCGTGC
OsGATA8-cr2 CCGATC--GAAGGTGCGTGCGTGC
PAM
Target
d
Maximum tillering stage
Mature stage
ZH11
OsGATA8-cr4
ZH11
OsGATA8-cr4
LN
HN
e
P = 4.7×10-4
PTNR
ZH11
OsGATA8-cr4
f
Total number of tillers
Number of productive tillers
#
LN
HN
Δ=-53.3%
Δ=-33.6%
Δ=-44.1%
Δ=-25.1%
ZH11
OsGATA8-cr4
ZH11
OsGATA8-cr4
g
Proportion of productive tillers (%)
LN
HN
P = 1.8×10-5
P = 1.8×10-5
h
Non-productive tillers per plant
LN
HN
P = 2.4×10-3
P = 2.5×10-6

Supplementary Figure 3: OsGATA8 negatively regulates the proportion of productive tillers in rice.

(a) Subcellular localization of the OsGATA8-GFP fusion protein in rice protoplasts. D53-mCherry is used as a nuclear marker. Scale bars, 8 μm. The results are representative of three independent experiments.
(b) Expression analysis of *OsGATA8* in various rice tissues after flowering by quantitative RT-PCR. Values represent mean ± SD derived from three individual plants.
(c) Mutations in the four *OsGATA8* CRISPR knockout lines (*OsGATA8*-cr1, -cr2, and -cr3 in the Nipponbare (Nip) background and *OsGATA8*-cr4 in the Zhonghua 11 (ZH11) background). Black bars: the coding region; White bars: the UTRs; Red lines: locations of the editing targets.
(d) Phenotypes of ZH11 and the *OsGATA8*-cr4 line under LN and HN conditions. HN, high nitrogen (300 kg/ha net nitrogen); LN, low nitrogen (75 kg/ha net nitrogen). Scale bars, 20 cm.
(e) PTNR of ZH11 and the *OsGATA8*-cr4 line. n = 20 plants. PTNR, productive-tiller-number ratio (productive tiller number under LN condition / productive tiller number under HN condition).
(f) The numbers of total number of tillers and productive tillers of ZH11 and the *OsGATA8*-cr4 line under LN and HN conditions. "Δ" represents the percentage difference compared with the total number of tillers. n = 20 plants.
(g-h) The proportion of productive tillers (PT%) **(g)** and non-productive tillers **(h)** of ZH11 and the *OsGATA8*-cr4 line under LN and HN conditions. n = 20 plants.
In **e**, **g**, **h**, P values were calculated with two-tailed Student's t test. In **f**, different letters indicate significant differences ($P < 0.05$, one-way ANOVA, Duncan's new multiple range test); for P values, see Supplementary Data 3. In **e** and **f-h**, box plots denote the 25th percentile, the median, and the 75th percentile, with minimum to maximum whiskers.

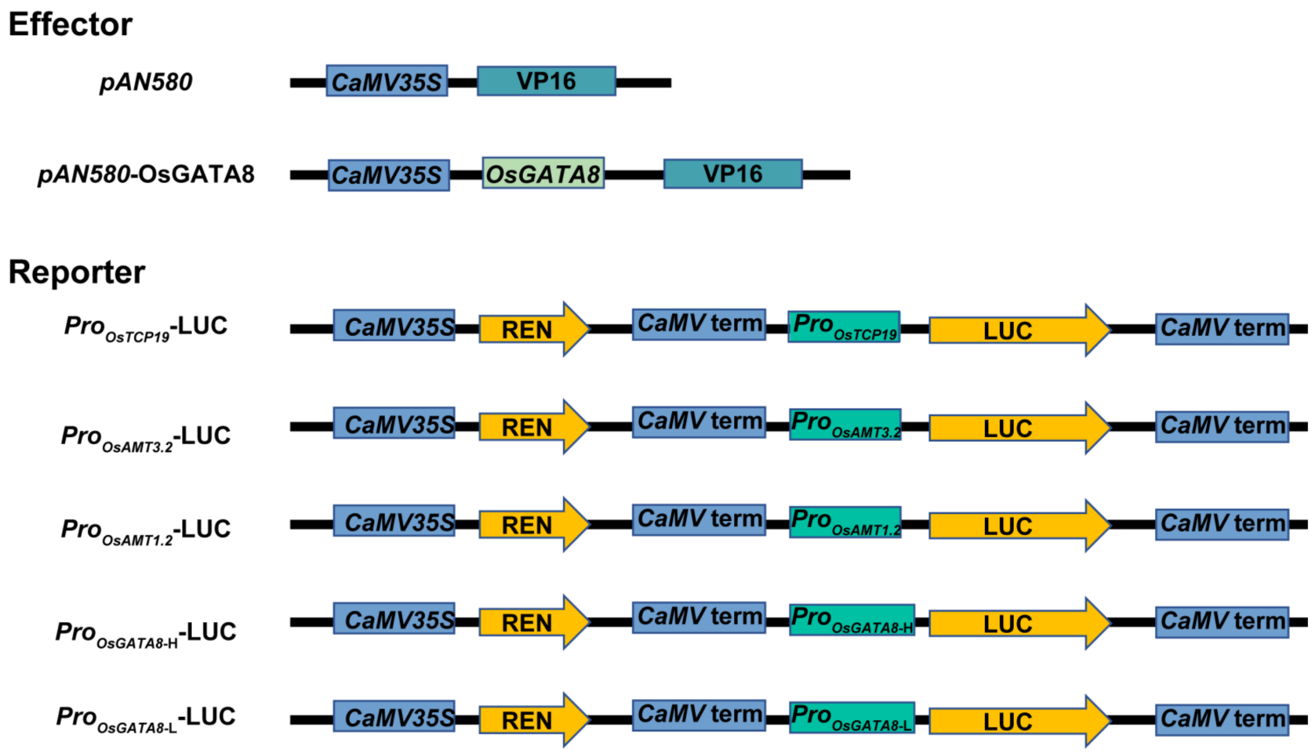

Supplementary Figure 4: Schematic diagrams of the effector and reporter plasmids used in the luciferase assays in rice protoplasts.

REN represents the *Renilla* luciferase; LUC represents the firefly luciferase.

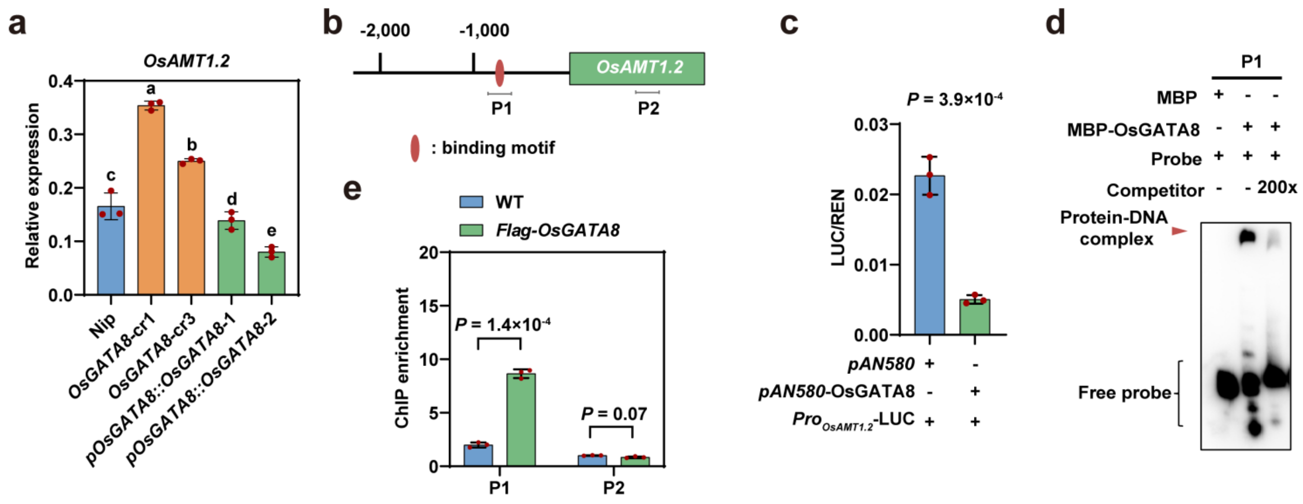

Supplementary Figure 5: OsGATA8 directly represses the expression of the ammonium transporter gene *OsAMT1.2*

(a) Relative expression of *OsAMT1.2* in the *OsGATA8* knockout lines and the *OsGATA8*

overexpression lines with its native promoter. Total RNA was extracted from roots of two-week-old seedlings. Each analysis was repeated with root tissues from three individual rice seedlings. Data are presented as mean ± SD.

(b) Schematic diagram of *OsAMT1.2* with the promoter and transcribed region. Horizontal bars indicate the location of the probe used in the electrophoretic mobility shift assay (EMSA). P1 correspond to the predicted OsGATA8 binding motif, while P2 is a negative control in the coding region without predicted OsGATA8 binding motif.

(c) Luciferase assays in rice protoplasts on the effect of OsGATA8 on the transcription of *OsAMT1.2*. Values represent mean ± SD derived from three independent samples of rice protoplasts.

(d) An EMSA testing the binding strength of OsGATA8 to the predicted binding motifs in *OsAMT1.2* promoter using probes as shown in **b**. The results are representative of three independent experiments.

(e) ChIP-qPCR assay of the interaction between OsGATA8 and the promoter of *OsAMT1.2* in the seedlings of *p35S::Flag-OsGATA8* transgenic plants. Values represent mean ± SD derived from three independent samples.

In **a**, different letters indicate significant differences ($P < 0.05$, one-way ANOVA, Duncan's new multiple range test); for P values, see Supplementary Data 4. In **c**, **e**, P values were calculated with two-sided Student's t test (**, $P < 0.01$).

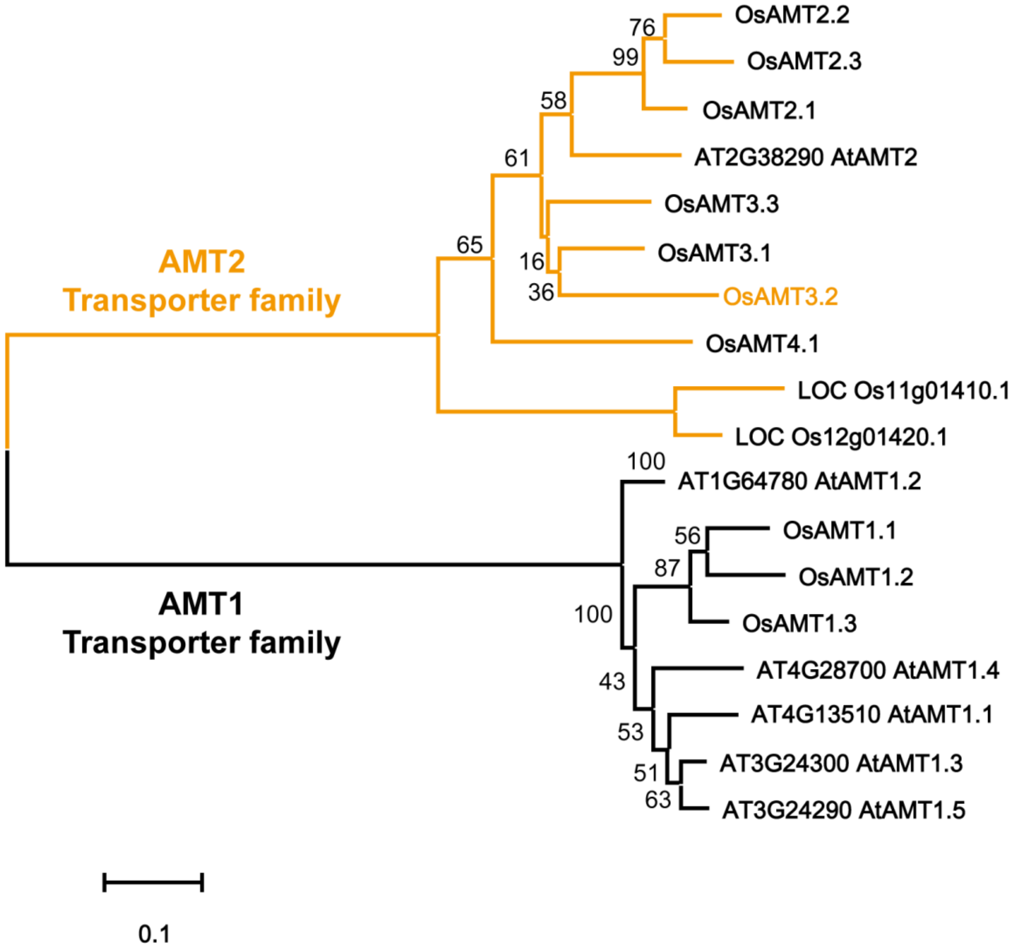
OsAMT2.2
OsAMT2.3
OsAMT2.1
AT2G38290 AtAMT2
OsAMT3.3
OsAMT3.1
OsAMT3.2
OsAMT4.1
LOC Os11g01410.1
LOC Os12g01420.1
AT1G64780 AtAMT1.2
OsAMT1.1
OsAMT1.2
OsAMT1.3
AT4G28700 AtAMT1.4
AT4G13510 AtAMT1.1
AT3G24300 AtAMT1.3
AT3G24290 AtAMT1.5
AMT2
Transporter family
AMT1
Transporter family
76
99
58
61
65
16
36
100
56
87
100
43
53
51
63
0.1

Supplementary Figure 6: Phylogenetic analysis of AMTs in rice and *Arabidopsis*. The amino acid sequences of AMTs in rice and their homologs in *Arabidopsis thaliana* were aligned by the MEGA7 software.

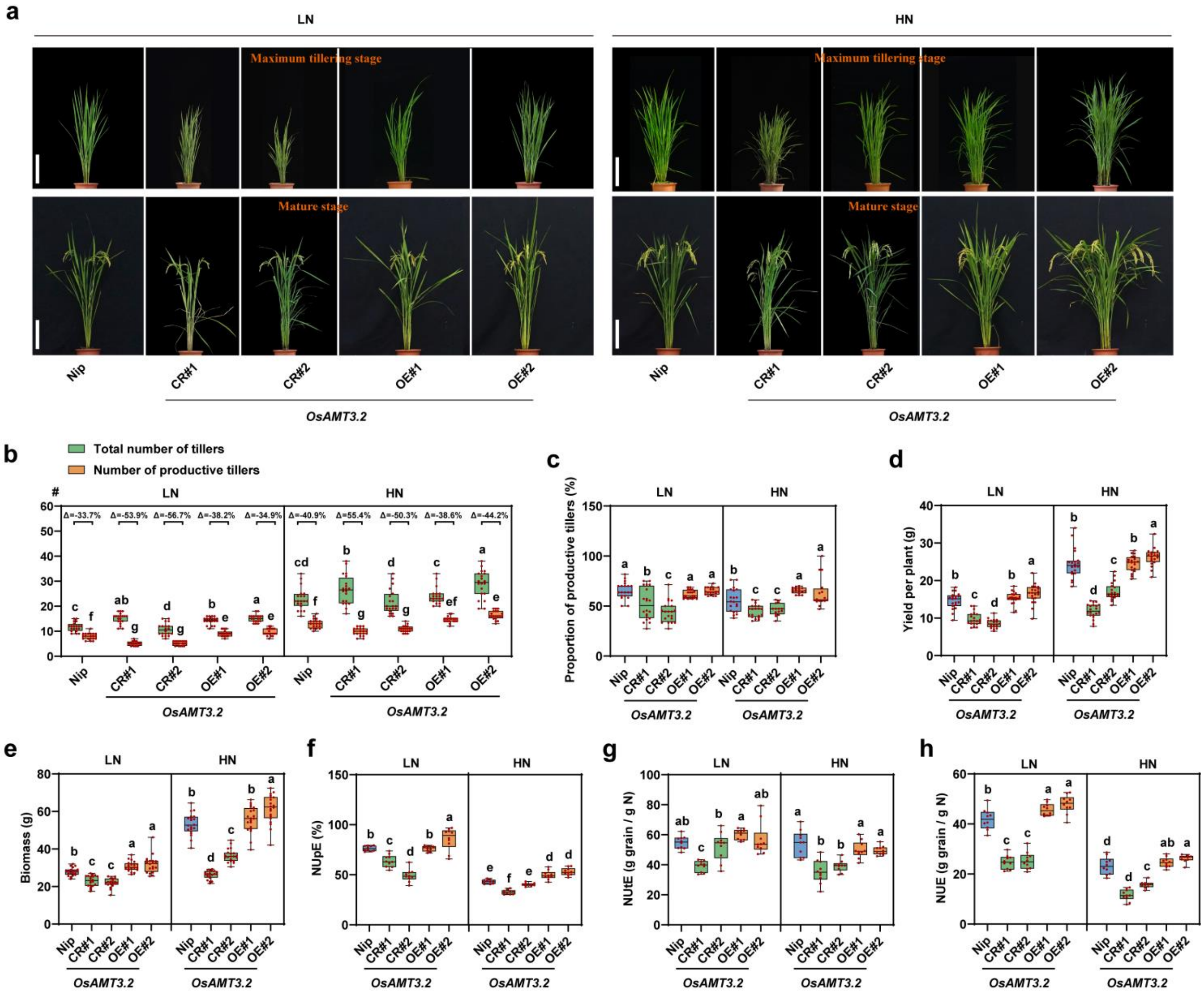

Supplementary Figure 7: *OsAMT3.2* positively regulates rice yield traits and NUE.

(a) Phenotypes of the *OsAMT3.2* knockout and overexpression lines under LN and HN conditions at the maximum tillering stage and mature stage. Scale bars, 20 cm; LN, low nitrogen (75 kg/ha net nitrogen); HN, high nitrogen (300 kg/ha net nitrogen).

(b-c) The numbers of total number of tillers, productive tillers, and the proportion of productive tillers (PT%) of the genotypes in **a** under LN and HN conditions. "Δ" represents the percentage difference compared with the total number of tillers. n = 20 plants.

(d-e) The yield per plant and biomass of *OsAMT3.2* knockout and overexpression lines under LN

and HN conditions at the mature stage (n = 20 plants).

(f-h) Nitrogen uptake efficiency (NUpE), Nitrogen utilization efficiency (NUtE) and Nitrogen use efficiency (NUE) of *OsAMT3.2* knockout and overexpression lines under LN and HN conditions at mature stage (n = 10 plants).

Nitrogen uptake efficiency (NUpE) was calculated by dividing the total nitrogen in shoot by the amount of nitrogen fertilizer. Nitrogen utilization efficiency (NUtE) was calculated by dividing the dry shoot biomass or grain yield by the total nitrogen in shoot. NUE = NUpE × NUtE[3]. In **b-h**, different letters indicate significant differences (P < 0.05, one-way ANOVA, Duncan's new multiple range test); for P values, see Supplementary Data 5; box plots denote the 25th percentile, the median, and the 75th percentile, with minimum to maximum whiskers.

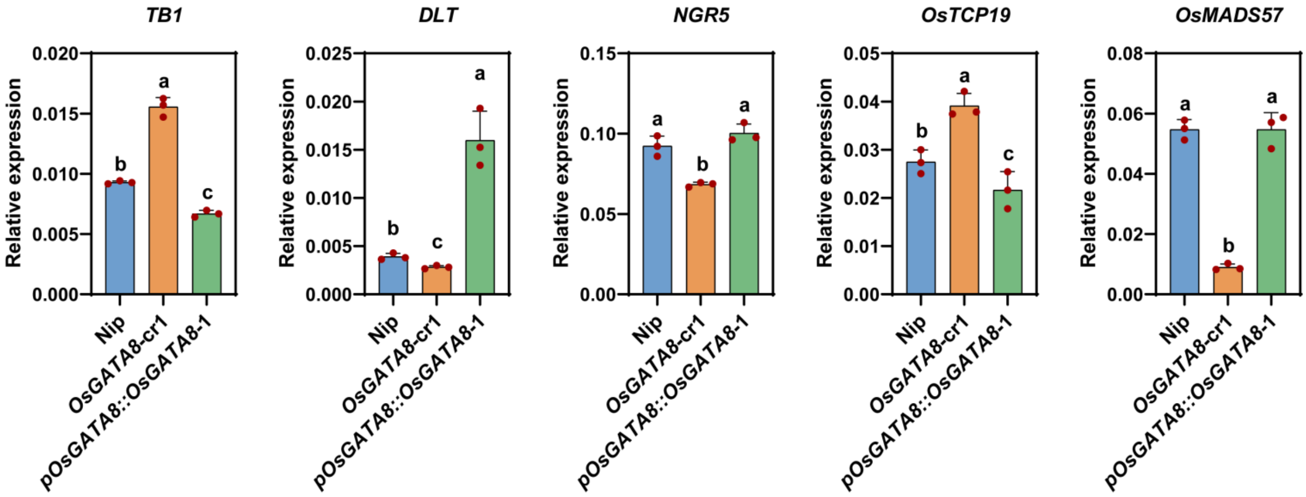

Supplementary Figure 8: OsGATA8 affects the expression of genes related to tillering.

The relative expression of genes that have been reported to be involved in rice tillering formation and nitrogen response of rice tiller buds in WT, *OsGATA8*-cr1 and *pOsGATA8::OsGATA8*-1 overexpression plants. Values represent mean ± SD derived from tiller buds of three individual plants. Different letters indicate significant differences (P < 0.05, one-way ANOVA, Duncan's new multiple range test); for P values, see Supplementary Data 6. Total RNA was extracted from tiller buds at the maximum tillering stage.

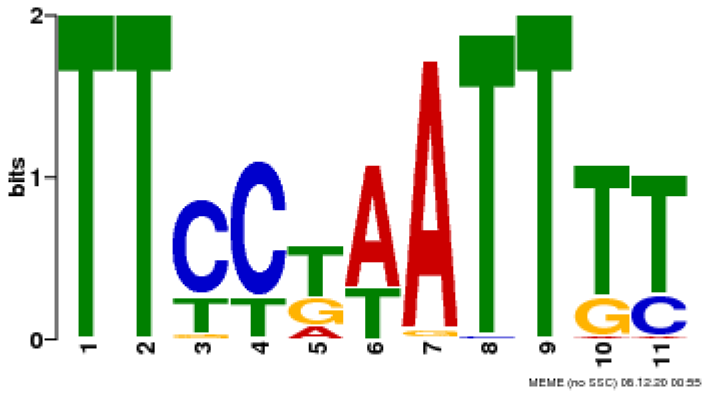

Supplementary Figure 9: Binding motif of OsGATA8 based on DAP-Seq assay

The significant motifs (E-value ≤ 0.05) found by the programs MEME, DREME, and CentriMo; clustered by similarity and ordered by E-value (MEME suit: http://meme-suite.org/).

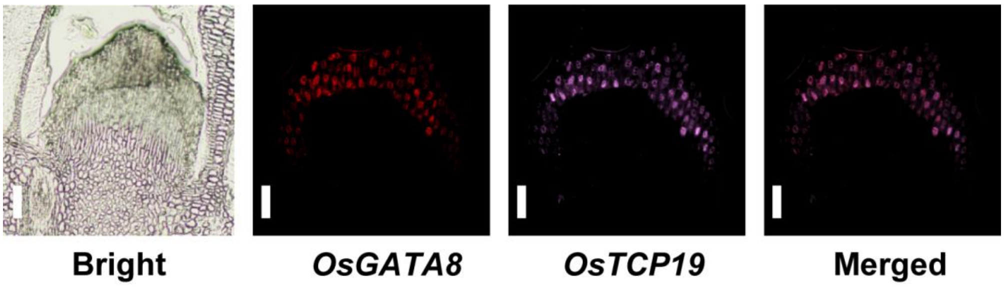

Supplementary Figure 10: *OsGATA8* and *OsTCP19* display similar expression pattern in the rice shoot apex.

Fluorescence *in situ* hybridization (FISH) (Bar = 25 μm) of *OsGATA8* and *OsTCP19* in rice SAM (Shoot Apical Meristem). The RNA of *OsGATA8* fluorescence-labeled with Cy5 and *OsTCP19* fluorescence-labeled with Cy3. The experiment was repeated three times with similar results.

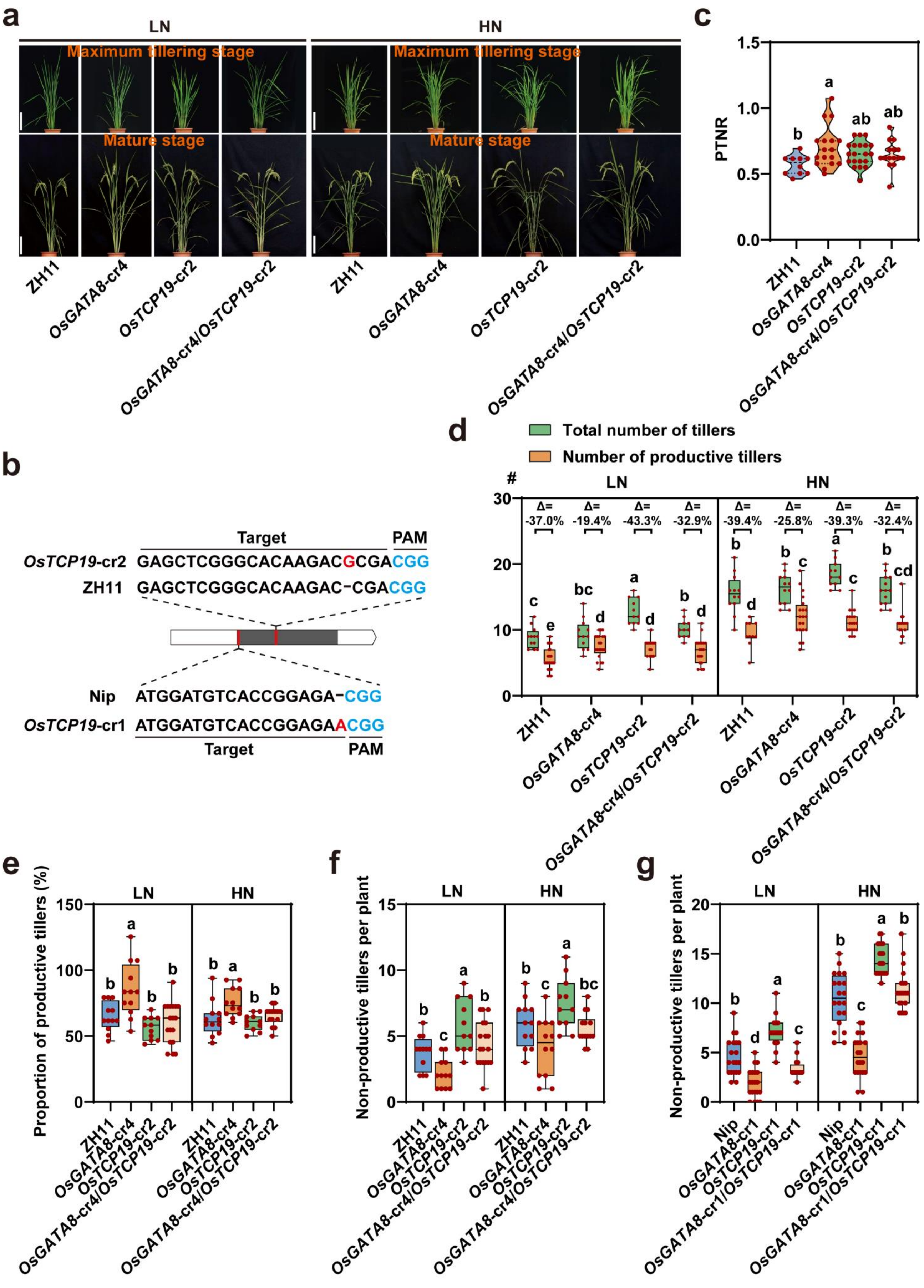
a
LN
HN
Maximum tillering stage
Mature stage
ZH11
OsGATA8-cr4
OsTCP19-cr2
OsGATA8-cr4/OsTCP19-cr2
b
Target
PAM
OsTCP19-cr2 GAGCTCGGGCACAAGACGCGACGG
ZH11 GAGCTCGGGCACAAGAC-CGACGG
Nip ATGGATGTCACCGGAGA-CGG
OsTCP19-cr1 ATGGATGTCACCGGAGAACGG
c
PTNR
d
Total number of tillers
Number of productive tillers
#
Δ= -37.0%
Δ= -19.4%
Δ= -43.3%
Δ= -32.9%
Δ= -39.4%
Δ= -25.8%
Δ= -39.3%
Δ= -32.4%
e
Proportion of productive tillers (%)
f
Non-productive tillers per plant
g
Nip
OsGATA8-cr1
OsTCP19-cr1
OsGATA8-cr1/OsTCP19-cr1

Supplementary Figure 11: *OsGATA8* acts upstream of *OsTCP19*, which promotes the formation of non-productive tillers.

(a) Phenotypes of Zhonghua 11 (ZH11), *OsGATA8*-cr4, *OsTCP19*-cr2, and *OsGATA8*-cr4/*OsTCP19*-cr2 under LN and HN conditions at the maximum tillering stage and mature stage. Scale bars, 20 cm.

(b) Diagram showing mutations in the *OsTCP19* CRISPR knockout lines (*OsTCP19*-cr1 in the Nipponbare (Nip) background and *OsTCP19*-cr2 in the ZH11 background). Black bars: the coding region of *OsTCP19*; White bars: the UTRs of *OsTCP19*; Red lines: the mutation sites of *OsTCP19*. The guide RNA targeting site and protospacer adjacent motif (PAM) are indicated. Inserted bases are marked in red.

(c) PTNR of the genotypes in **a** under LN and HN conditions. *n* = 10 plants. PTNR, productive-tiller-number ratio (productive tiller number under LN condition / productive tiller number under HN condition).

(d) The numbers of total number of tillers and productive tillers of the genotypes in **a** under LN and HN conditions. "Δ" represents the percentage difference compared with the total number of tillers. *n* = 12 plants.

(e-f) The proportion of productive tillers (PT%) and non-productive tillers of the genotypes in **a** under LN and HN conditions. *n* = 12 plants.

(g) The non-productive tillers of the genotypes in **Fig. 3e** under LN and HN conditions. *n* = 20 plants.

In **c-g**, different letters indicate significant differences ($P < 0.05$, one-way ANOVA, Duncan's new multiple range test); for P values, see Supplementary Data 7. LN, low nitrogen (75 kg/ha net nitrogen); HN, high nitrogen (300 kg/ha net nitrogen). In **c**, the bars in the violin plots represent the 25th percentile, the median, and the 75th percentile, respectively; in **d-g**, box plots denote the 25th percentile, the median, and the 75th percentile, with minimum to maximum whiskers.

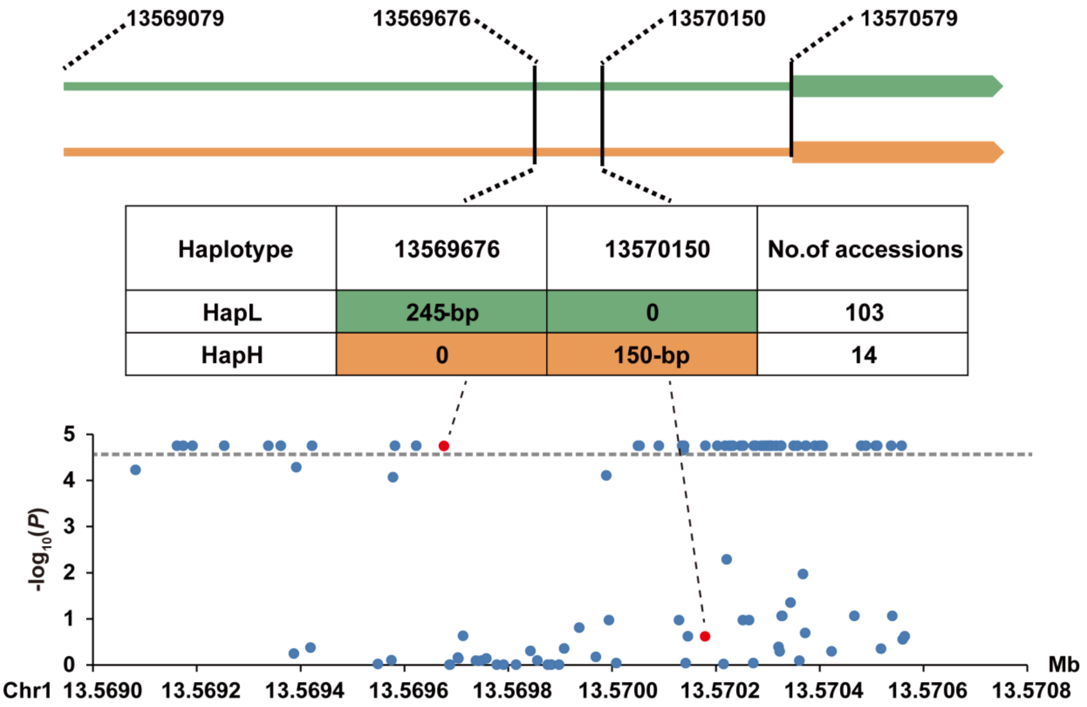

Supplementary Figure 12: Natural variations within the *OsGATA8* promoter.

Structure of *OsGATA8* (top) and association mapping with its promoter variations (bottom). *P* values were determined under the mixed linear model and implemented in Tassel 5. Dots above dashed line indicate variations significantly associated with PTNR. Red dots connected with the dashed lines indicate two large indel that are associated with PTNR. x axis, physical position of *OsGATA8* promoter.

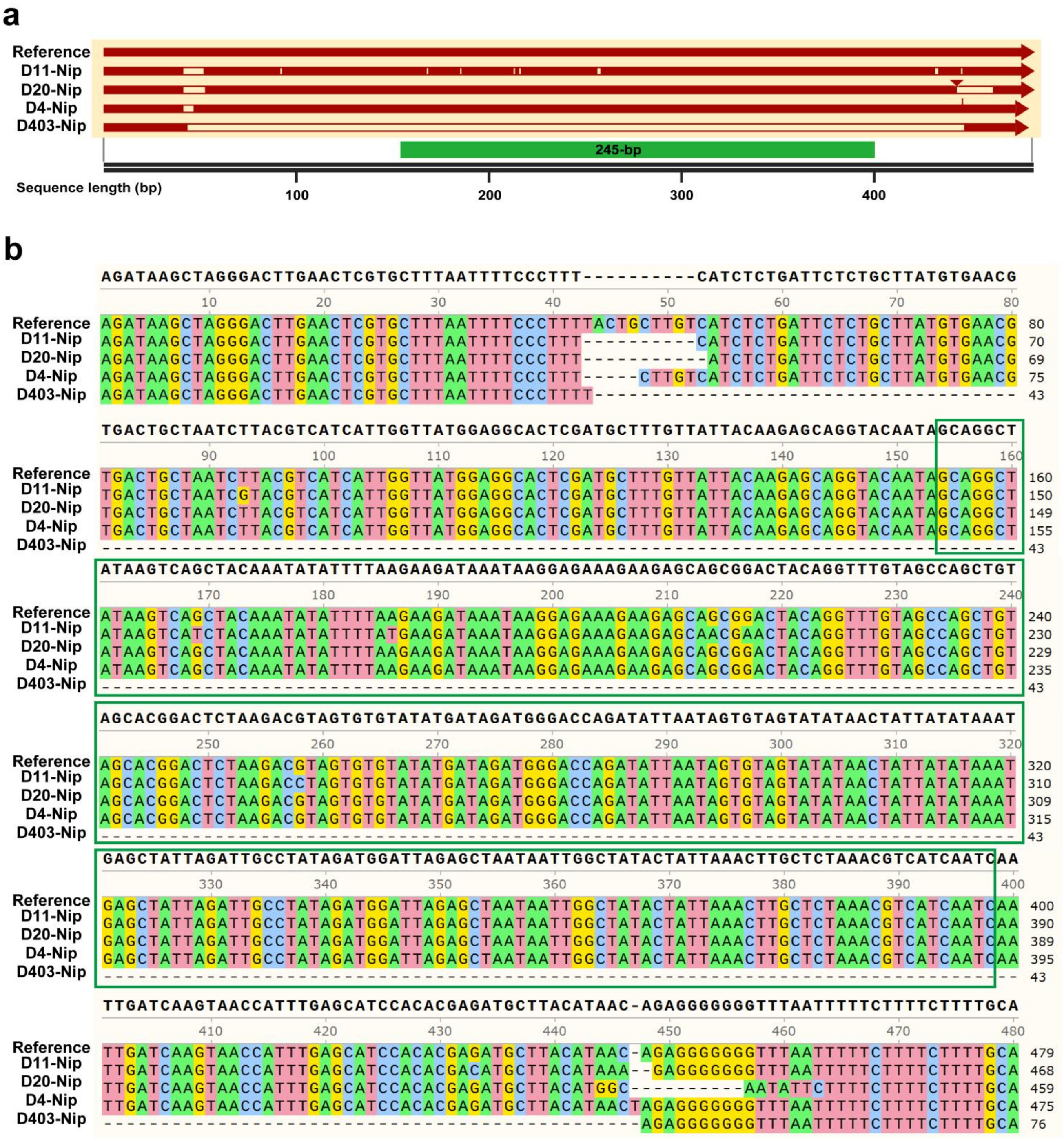

Supplementary Figure 13: Alignment of the sequences near the 245-bp variable region within the *OsGATA8* promoter in the four homozygous deletion lines.

(a) Schematic representation of four homozygous lines near the 245-bp variable region of the *OsGATA8* promoter. The green bar marks the location of the 245-bp sequence.

(b) Alignment of the nucleotide sequences near the 245-bp variant region within the *OsGATA8* promoter in the four homozygous deletion lines. The 245-bp sequence is indicated with the green box.

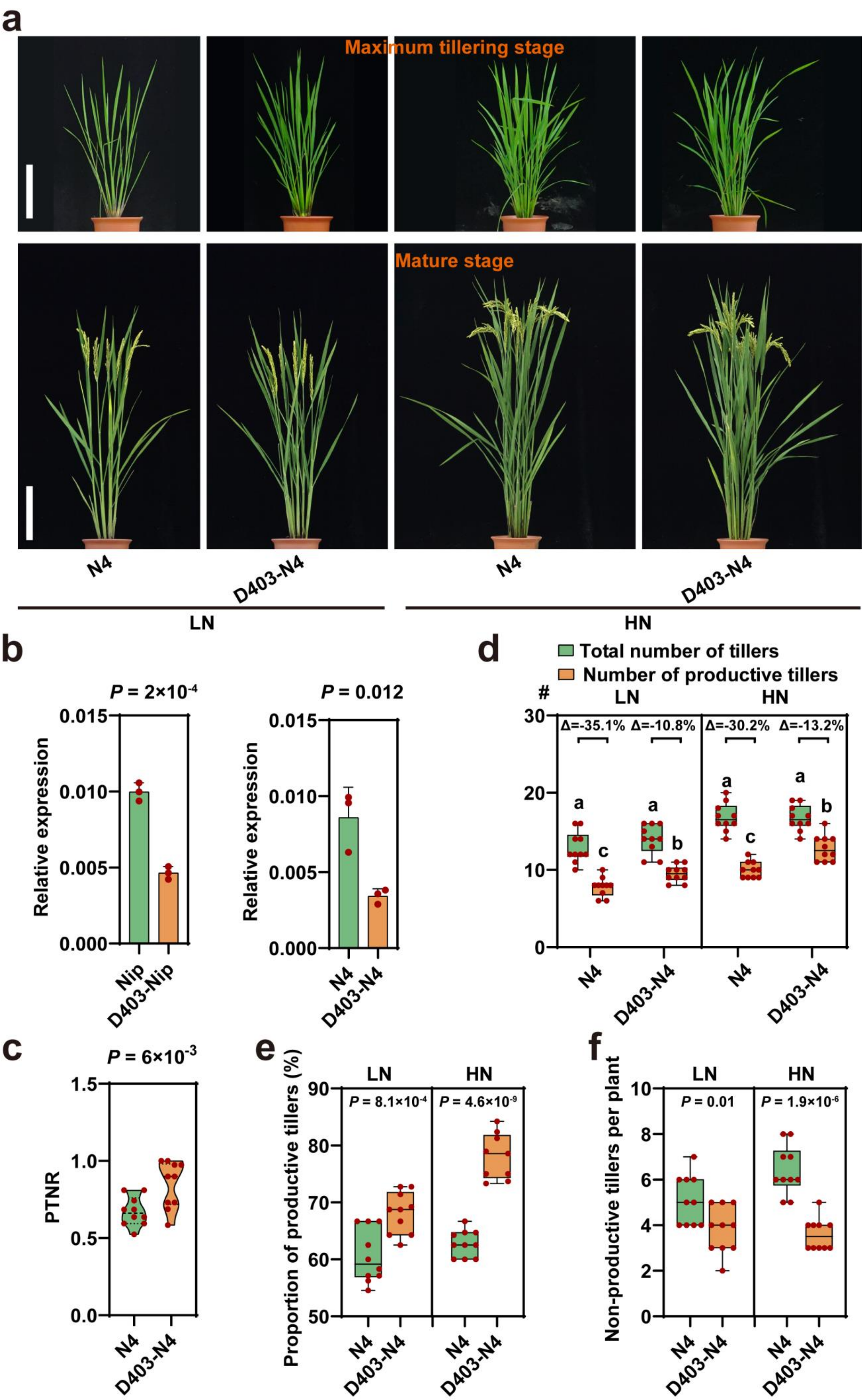
a
Maximum tillering stage
Mature stage
N4
D403-N4
N4
D403-N4
LN
HN
b
P = 2×10-4
Relative expression
Nip
D403-Nip
P = 0.012
Relative expression
N4
D403-N4
c
P = 6×10-3
PTNR
N4
D403-N4
d
Total number of tillers
Number of productive tillers
#
LN
HN
Δ=-35.1%
Δ=-10.8%
Δ=-30.2%
Δ=-13.2%
N4
D403-N4
N4
D403-N4
e
Proportion of productive tillers (%)
LN
HN
P = 8.1×10-4
P = 4.6×10-9
N4
D403-N4
N4
D403-N4
f
Non-productive tillers per plant
LN
HN
P = 0.01
P = 1.9×10-6
N4
D403-N4
N4
D403-N4

Supplementary Figure 14: A 245-bp deletion in the promoter of *OsGATA8* promotes NUE in rice.

(a) Phenotypes of D403-N4 at the maximum tillering stage and the mature stage compared with N4 under LN and HN conditions. Scale bars, 20 cm.

(b) Relative expression of *OsGATA8* in the D403 lines in the background of Nip and N4. Values represent mean ± SD derived from three individual rice seedlings. Total RNA was extracted from two-week-old seedlings.

(c) PTNR of D403-N4 line under LN and HN conditions. *n* = 10 plants. PTNR, productive-tiller-number ratio (productive tiller number under LN condition / productive tiller number under HN condition).

(d) The numbers of total number of tillers and productive tillers of ZH11 and the *OsGATA8*-cr4 line under LN and HN conditions. "Δ" represents the percentage difference compared with the total number of tillers. *n* = 10 plants.

(e-f) The proportion of productive tillers (PT%) **e** and non-productive tillers **f** of ZH11 and the *OsGATA8*-cr4 line under LN and HN conditions. *n* = 10 plants.

LN, low nitrogen (75 kg/ha net nitrogen); HN, high nitrogen (300 kg/ha net nitrogen). In **b, c**, **e**, **f**, *P* values were calculated with two-tailed Student's *t* test. In **d**, different letters indicate significant differences ($P < 0.05$, one-way ANOVA, Duncan's new multiple range test); for *P* values, see Supplementary Data 8. In **c**, the bars in the violin plots represent 25th percentile, median and 75th percentile; in **d**-**f**, box plots denote the 25th percentile, the median, and the 75th percentile, with minimum to maximum whiskers.

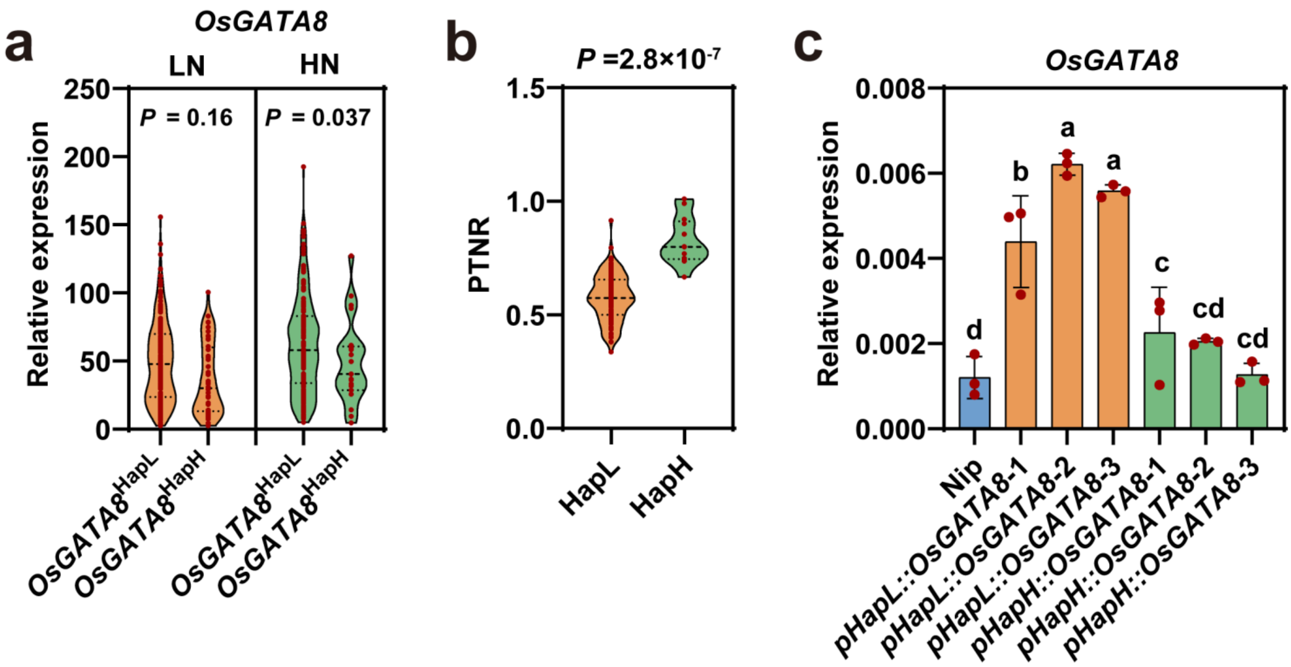

Supplementary Figure 15: The elite haplotype of the *OsGATA8* promoter exhibits reduced expression and increased NUE.

(a) Relative expression of *OsGATA8* (FPKM) in 175 cultivars selected from Rice 3K under LN and HN conditions. LN, low nitrogen (75 kg/ha net nitrogen); HN, high nitrogen (300 kg/ha net nitrogen).

(b) PTNR of 117 rice accessions under LN and HN conditions. PTNR, productive-tiller-number ratio (productive tiller number under LN condition / productive tiller number under HN condition).

(c) Relative expression of *OsGATA8* in Nipponbare (Nip) and the *OsGATA8* transgenic lines with the HapL or the HapH promoter (*pHapL::OsGATA8* and *pHapH::OsGATA8*). RNA used for qRT-PCR in **a** and **c** was extracted from the rice seedlings. Values represent mean ± SD derived from three individual rice seedlings.

In **a** and **b**, *P* values were calculated with one-tailed Student's *t* test. In **c**, different letters indicate significant differences ($P < 0.05$, one-way ANOVA, Duncan's new multiple range test); for *P* values, see Supplementary Data 9. In **a** and **b**, the bars within the violin plots represent the 25th percentile, the median, and the 75th percentile, respectively.

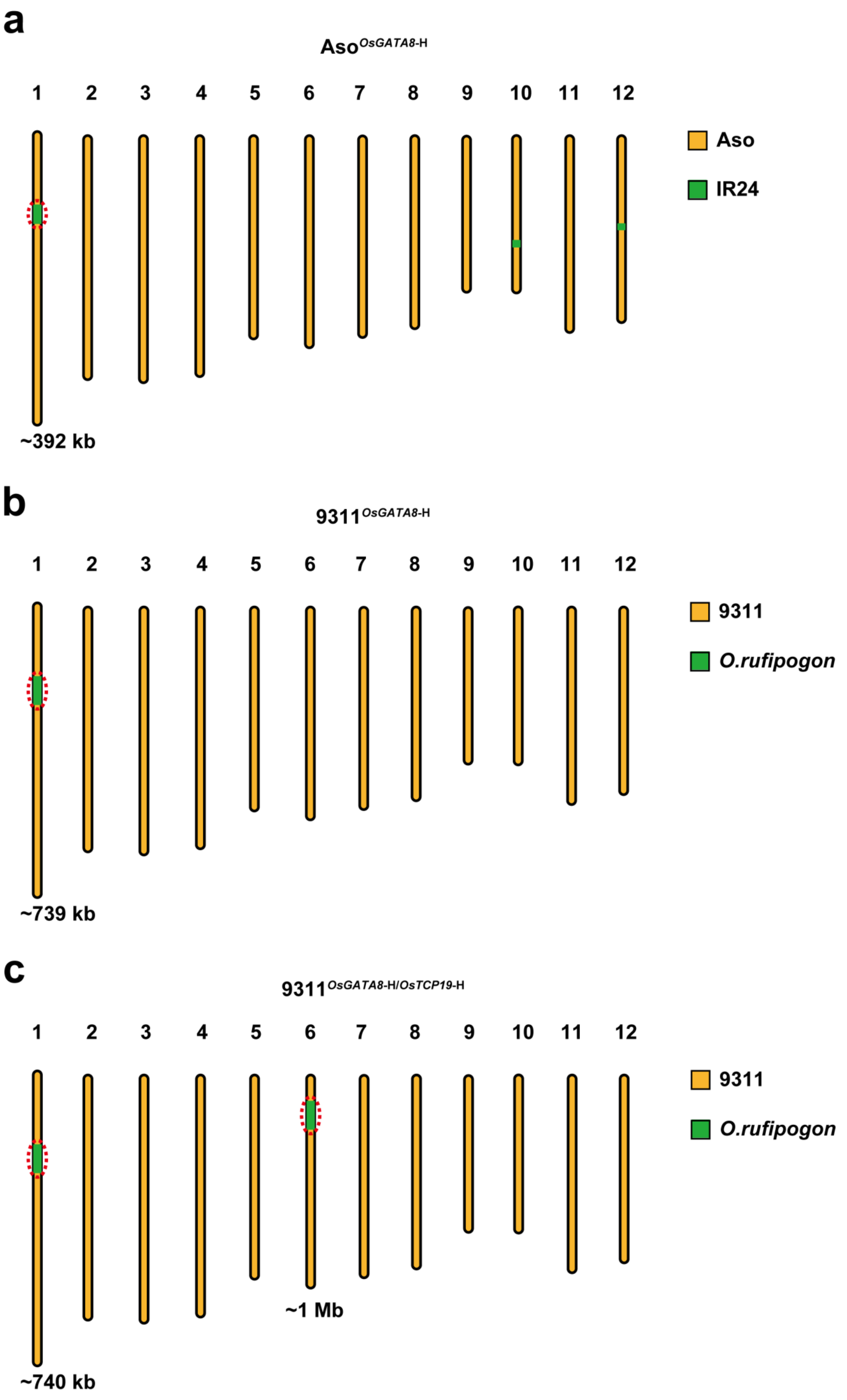
a
AsoOsGATA8-H
1 2 3 4 5 6 7 8 9 10 11 12
Aso
IR24
~392 kb
b
9311OsGATA8-H
1 2 3 4 5 6 7 8 9 10 11 12
9311
O.rufipogon
~739 kb
c
9311OsGATA8-H/OsTCP19-H
1 2 3 4 5 6 7 8 9 10 11 12
9311
O.rufipogon
~1 Mb
~740 kb

Supplementary Figure 16: Schematic diagram of NIL lines in this article.

(a) Schematic diagram of Aso$^{OsGATA8\text{-H}}$ under the Aso background.
(b-c) Schematic diagram of 9311$^{OsGATA8\text{-H}}$ and 9311$^{OsGATA8\text{-H}/OsTCP19\text{-H}}$ under the 9311 background.
The length of the introgressed DNA fragment (colored in green) is displayed at the bottom of each relevant chromosome.

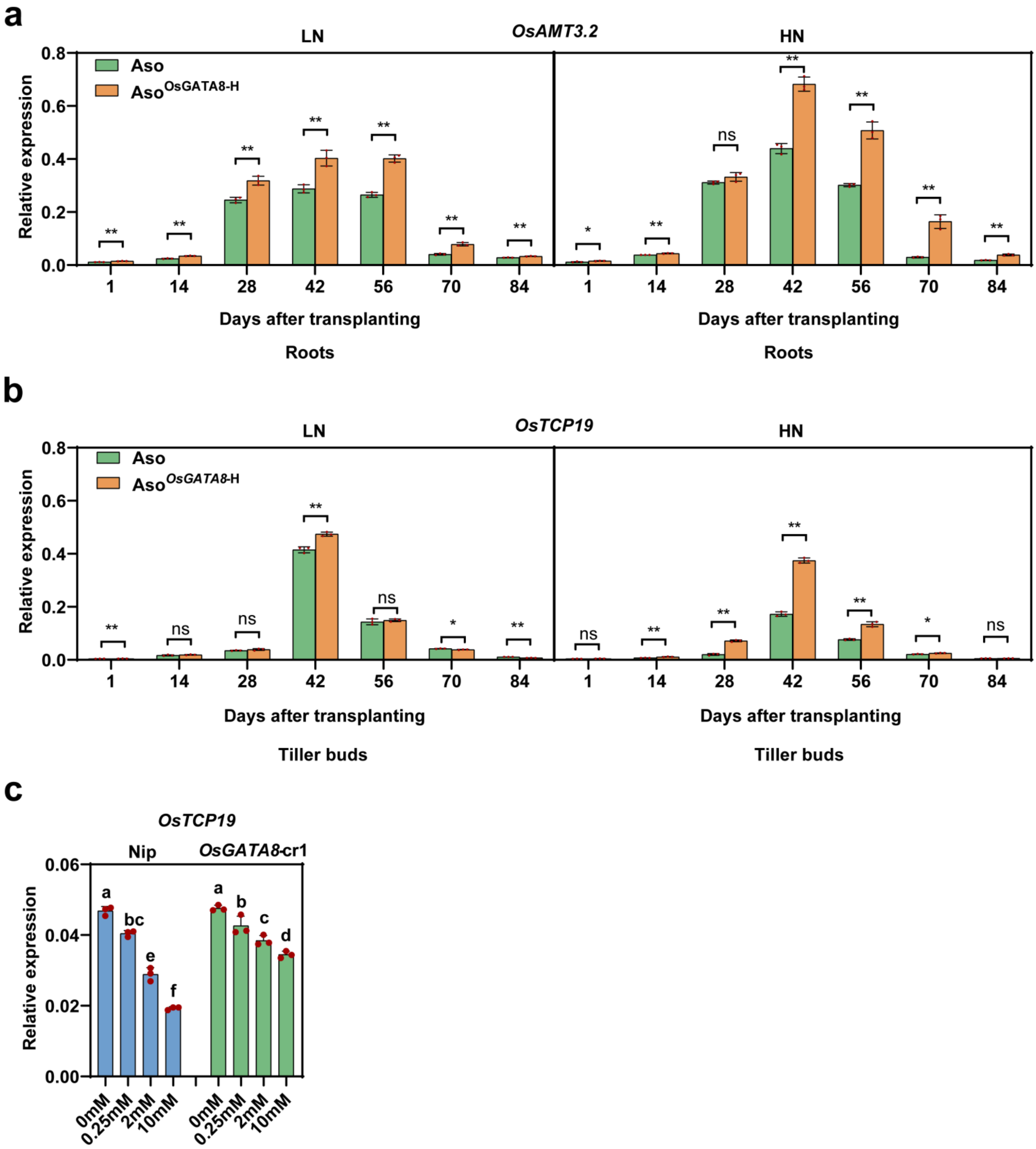

Supplementary Figure 17: OsGATA8 dynamically regulates the expression of *OsAMT3.2* and *OsTCP19* during rice growth and development

(a-b) Dynamic changes of *OsAMT3.2* and *OsTCP19* transcription levels in Aso and Aso$^{OsGATA8\text{-H}}$ roots and tiller buds with transplanting days. In **a**, total RNA was extracted from roots at different days after transplanting. in **b**, total RNA was extracted from tiller buds of rice at different days after transplanting; Values represent mean ± SD derived from root or tiller buds of three individual plants.
(c) Expression of *OsTCP19* in WT and *OsGATA8* knockout line under different nitrogen concentrations. Total RNA was extracted from shoot base of rice seedling. Values represent mean ± SD derived from shoot base of three individual rice seedlings.
In **a-b**, *P* values were calculated with two-tailed Student's *t* test (*, $P < 0.05$; **, $P < 0.01$); in **c**, different letters indicate significant differences ($P < 0.05$, one-way ANOVA, Duncan's new multiple range test); for *P* values, see Supplementary Data 10.

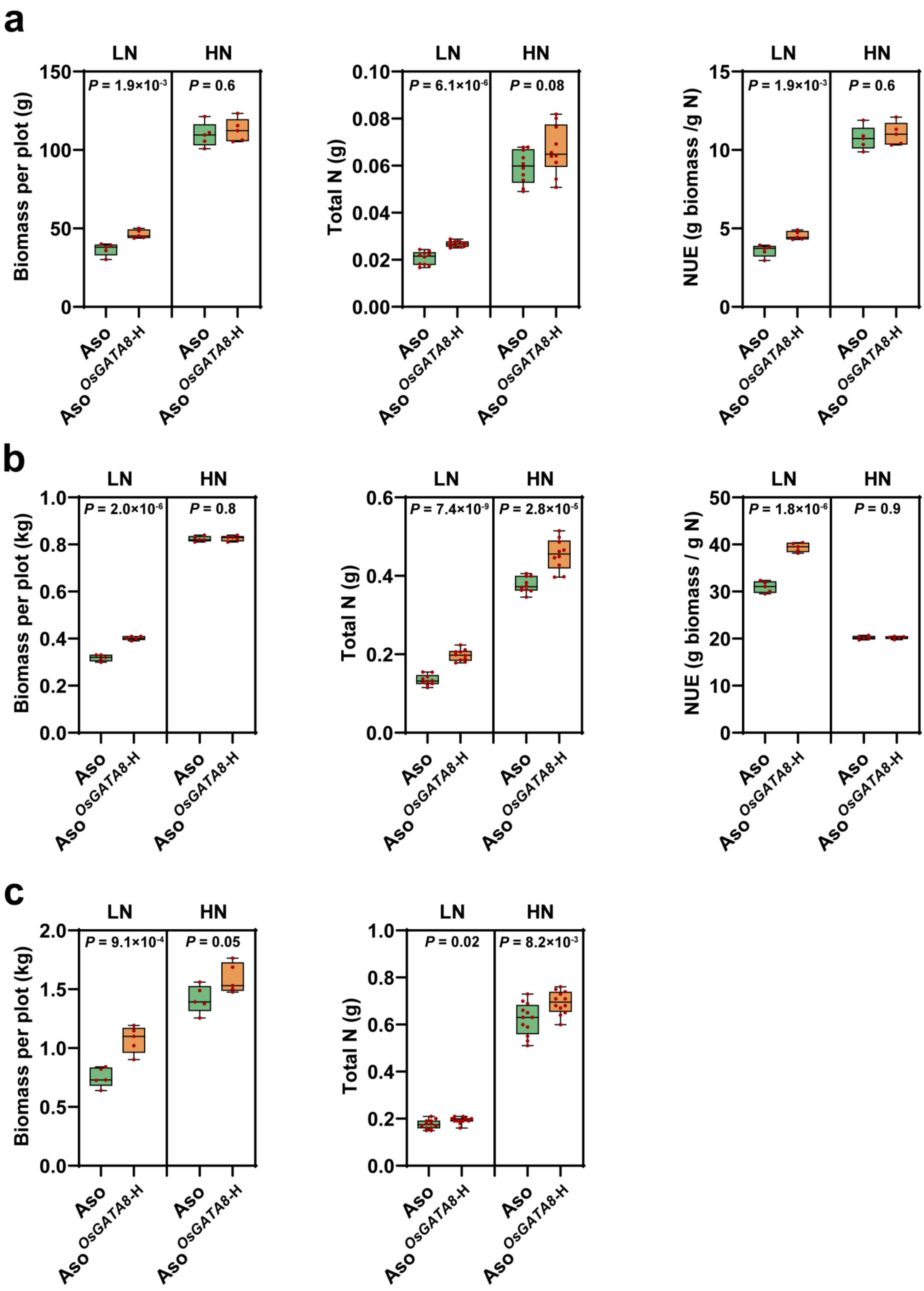
a
LN
HN
$P = 1.9\times10^{-3}$
$P = 0.6$
Biomass per plot (g)
Aso
Aso OsGATA8-H
$P = 6.1\times10^{-6}$
$P = 0.08$
Total N (g)
$P = 1.9\times10^{-3}$
$P = 0.6$
NUE (g biomass /g N)
b
$P = 2.0\times10^{-6}$
$P = 0.8$
Biomass per plot (kg)
$P = 7.4\times10^{-9}$
$P = 2.8\times10^{-5}$
Total N (g)
$P = 1.8\times10^{-6}$
$P = 0.9$
NUE (g biomass / g N)
c
$P = 9.1\times10^{-4}$
$P = 0.05$
Biomass per plot (kg)
$P = 0.02$
$P = 8.2\times10^{-3}$
Total N (g)

Supplementary Figure 18: OsGATA8 negatively regulates rice NUE.

(a) The biomass per plot (10 plants × 4 rows), total N per plant and NUE of *OsGATA8* NIL line at seedling stages under LN and HN conditions. Analysis in biomass of per plot was repeated with five plots; analysis in total N was repeated with ten individual plants; analysis in NUE was repeated with five plots.

(b) The biomass per plot (10 plants × 4 rows), total N per plant and NUE of *OsGATA8* NIL line at maximum tillering stages under LN and HN conditions. Analysis in biomass of per plot was repeated with five plots; analysis in total N was repeated with ten individual plants; analysis in NUE was repeated with five plots.

(c) The biomass per plot (10 plants × 4 rows) and total N per plant of *OsGATA8* NIL line at mature stage under LN and HN conditions. Analysis in biomass of per plot was repeated with five plots; analysis in total N was repeated with ten individual plants.

In **a-c**, box plots denote the 25th percentile, the median, and the 75th percentile, with minimum to maximum whiskers; different letters indicate significant differences ($P < 0.05$, one-way ANOVA, Duncan's new multiple range test); for *P* values, see Supplementary Data 11. LN, low nitrogen (75 kg/ha net nitrogen); HN, high nitrogen (300 kg/ha net nitrogen).

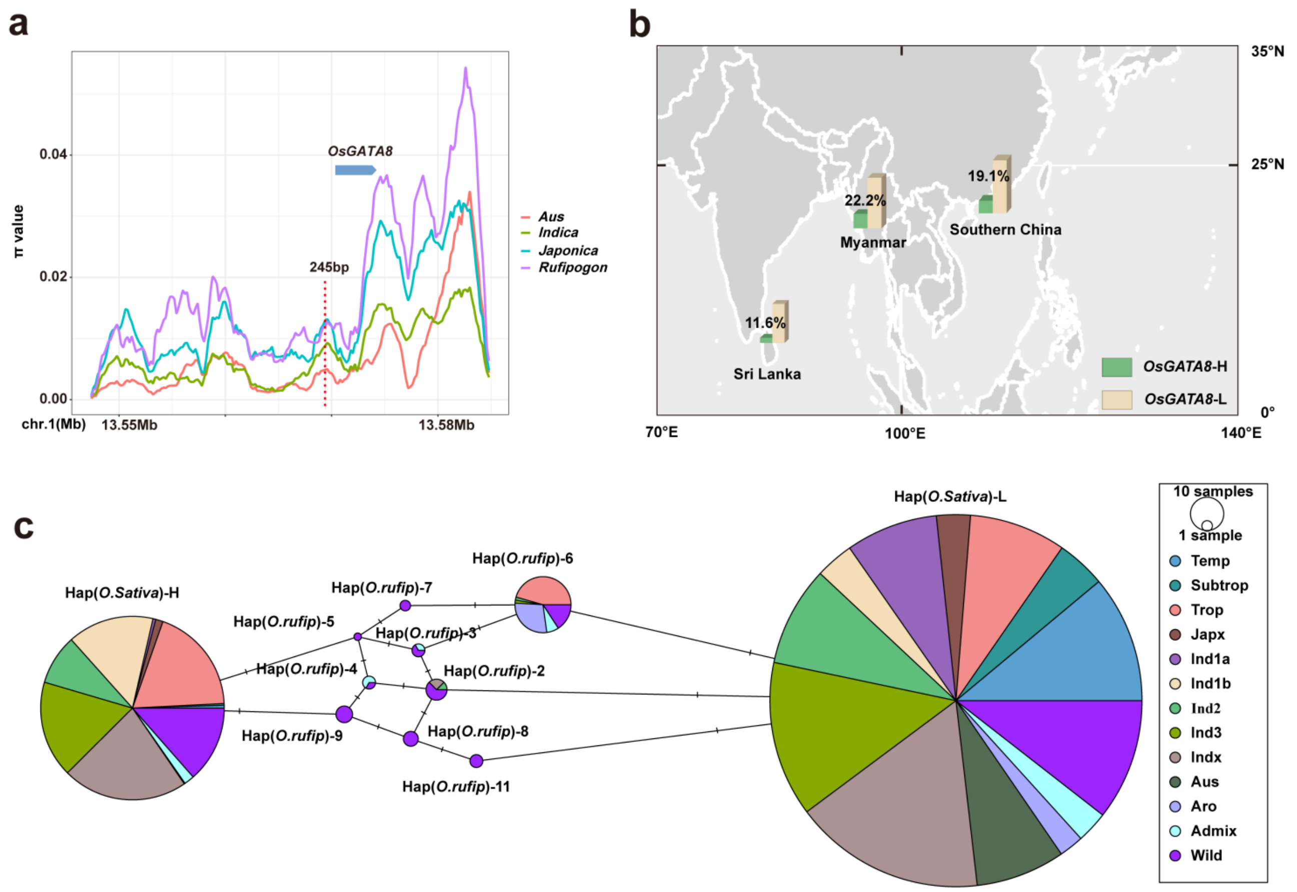

Supplementary Figure 19: *OsGATA8*-H is a rare and underutilized haplotype.

(a) Nucleotide diversity across *OsGATA8*. The red line indicates the physical location of the 245-bp variation within the *OsGATA8* promoter; the blue bar indicates the physical location of *OsGATA8* transcription region.

(b) The frequency of *OsGATA8*-H in wild rice populations from three different regions. The map was made using the 'maps' package in R[33].

(c) Haplotype network of *OsGATA8*.

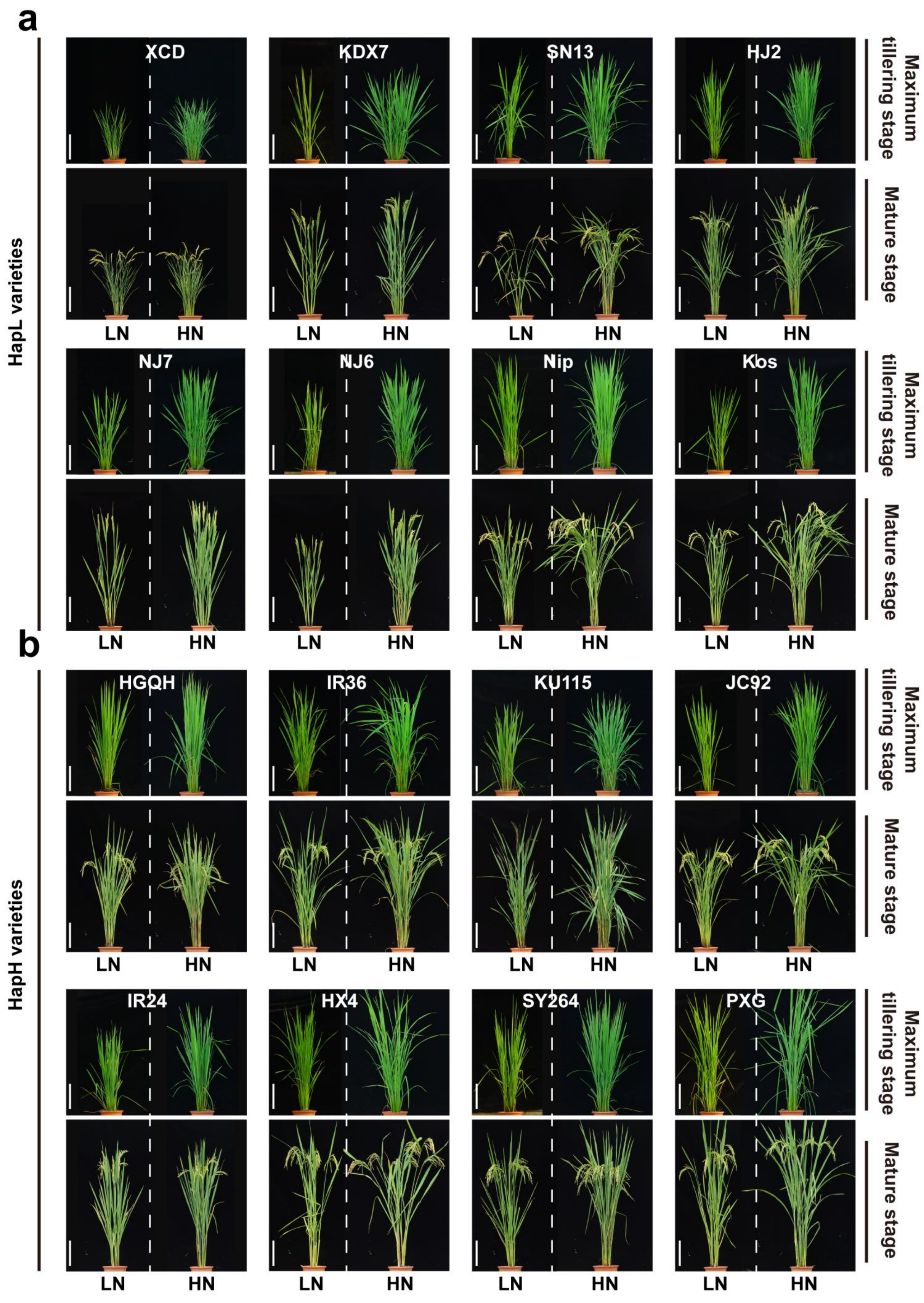
a
XCD
KDX7
SN13
HJ2
NJ7
NJ6
Nip
Kos
Maximum tillering stage
Mature stage
HapL varieties
LN
HN
b
HGQH
IR36
KU115
JC92
IR24
HX4
SY264
PXG
HapH varieties

Supplementary Figure 20: Phenotypes of *OsGATA8*-H/L varieties under LN and HN conditions at the maximum tillering stage and mature stage.

(a) Phenotypes of *OsGATA8*-L varieties at the maximum tillering and mature stages under LN and HN conditions.

(b) Phenotypes of *OsGATA8*-H varieties at the maximum tillering and mature stages under LN and HN conditions.

In **a**, **b**, scale bars, 20 cm. LN, low nitrogen (75 kg/ha net nitrogen); HN, high nitrogen (300 kg/ha net nitrogen).

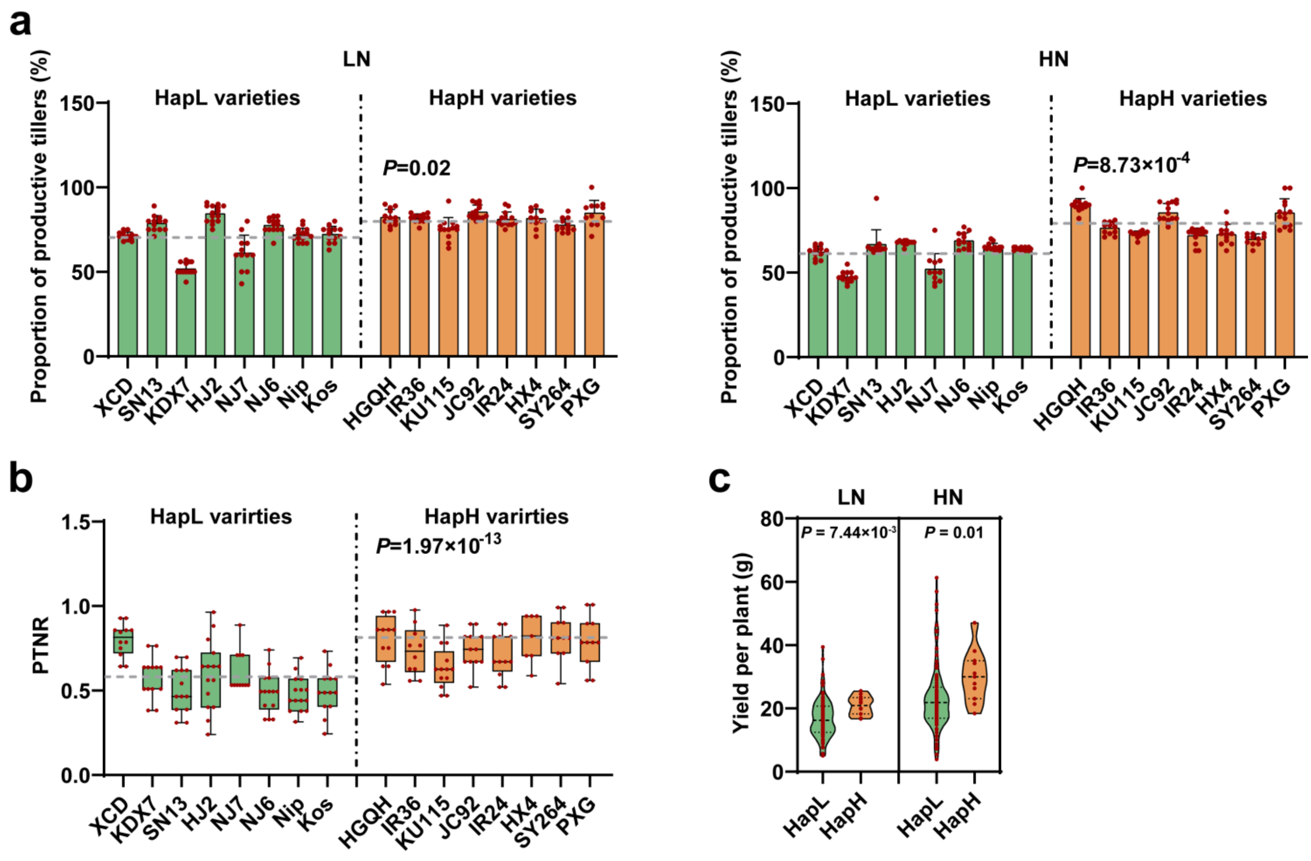

Supplementary Figure 21: Elite haplotype of *OsGATA8* confers higher proportion of productive tillers and yield.

(a-b) The proportion of productive tillers (PT%) and PTNR of *OsGATA8*-H/L varieties under LN and HN conditions. Data are presented as mean ± SD. The dotted lines represent the average values of two haplotypes varieties. LN, low nitrogen (75 kg/ha net nitrogen); HN, high nitrogen (300 kg/ha net nitrogen); PT%, proportion of productive tillers; PTNR, productive-tiller-number ratio (productive

tiller number under LN condition / productive tiller number under HN condition).

(c) The yield per plant of *OsGATA8*-H/L varieties. HapL varieties, n = 92 plants; HapL varieties, n = 12 plants.

In **a**-**c**, P values were calculated with two-tailed Student's t test. In **a**, **b**, n = 13 plants per cultivar × 8 cultivars = 104 plants. In **b**, box plots denote the 25th percentile, the median, and the 75th percentile, with minimum to maximum whiskers; in **c**, the bars within the violin plots represent the 25th percentile, the median, and the 75th percentile, respectively.

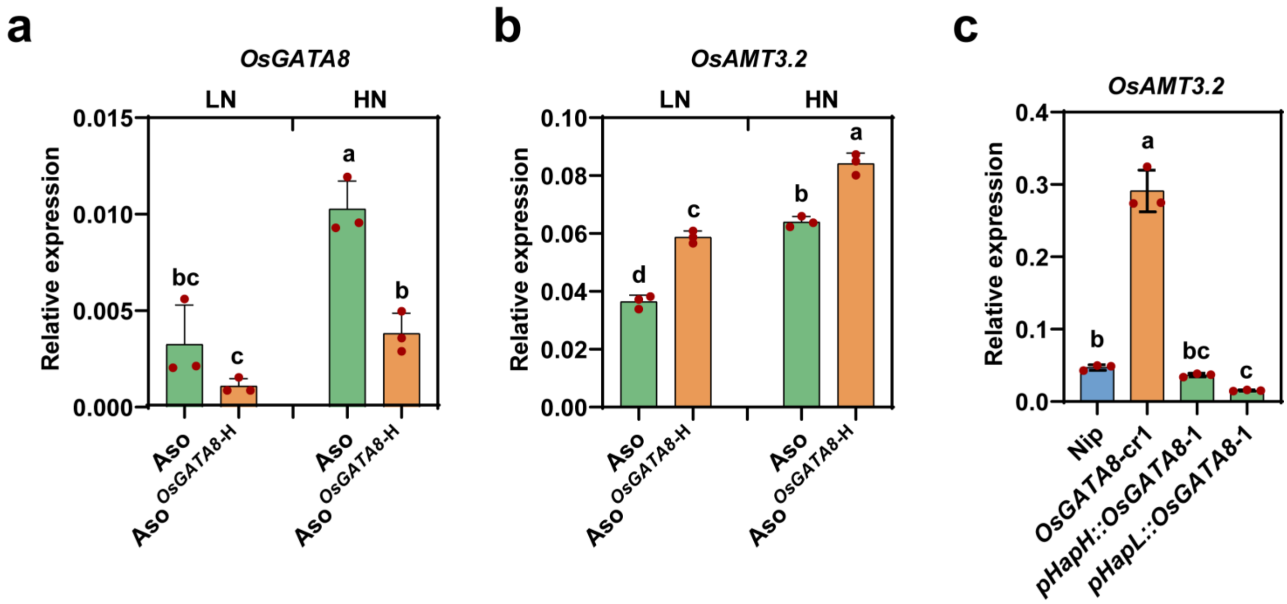

Supplementary Figure 22: Elite haplotype *OsGATA8*-H promotes rice yield and NUE.

(a-b) Relative expression of *OsGATA8* and *OsAMT3.2* in Aso and Aso$^{\textit{OsGATA8}\text{-H}}$ under LN and HN conditions. Total RNA was extracted from root tissue of two-week-old seedlings. Values represent mean ± SD derived from root tissues of three individual rice seedlings.

(c) Relative expression of *OsAMT3.2* in Nipponbare (Nip) and the *OsGATA8* transgenic lines with the HapL or the HapH promoter (*pHapL::OsGATA8* and *pHapH::OsGATA8*). Total RNA was extracted from the roots of two-week-old seedlings. Values represent mean ± SD derived from root tissues of three individual rice seedlings. LN, low nitrogen (0.2 mM NH_4NO_3); HN, high nitrogen (2.0 mM NH_4NO_3).

In **a**-**c**, different letters indicate significant differences (P < 0.05, one-way ANOVA, Duncan's new multiple range test), for P values, see Supplementary Data 13.

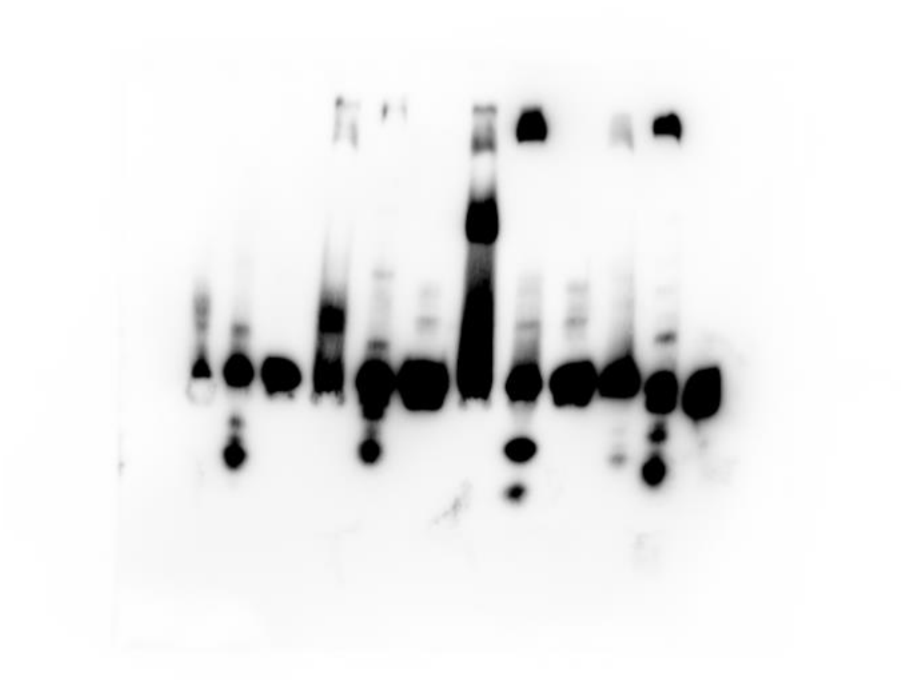

Supplementary Figure 23: Unprocessed EMSA blot of Supplementary Fig. 5d.
