# Supplementary material for: The elite haplotype OsGATA8-H coordinates nitrogen uptake and productive tiller formation in rice
Source: Nat Genet. 2024 Jun 13;56(7):1516–26. doi: 10.1038/s41588-024-01795-7 (PMC11250373; doi:10.1038/s41588-024-01795-7)
Supplement: Supplementary file 2 — Reporting Summary [file 41588_2024_1795_MOESM2_ESM.pdf]

nature portfolio

Corresponding author(s): Jianmin Wan, Chunming Wang

Last updated by author(s): May 6, 2024

Reporting Summary

Nature Portfolio wishes to improve the reproducibility of the work that we publish. This form provides structure for consistency and transparency in reporting. For further information on Nature Portfolio policies, see our Editorial Policies and the Editorial Policy Checklist.

Statistics

For all statistical analyses, confirm that the following items are present in the figure legend, table legend, main text, or Methods section.

n/a	Confirmed	
☐	☒	The exact sample size (*n*) for each experimental group/condition, given as a discrete number and unit of measurement
☐	☒	A statement on whether measurements were taken from distinct samples or whether the same sample was measured repeatedly
☐	☒	The statistical test(s) used AND whether they are one- or two-sided<br>*Only common tests should be described solely by name; describe more complex techniques in the Methods section.*
☒	☐	A description of all covariates tested
☒	☐	A description of any assumptions or corrections, such as tests of normality and adjustment for multiple comparisons
☐	☒	A full description of the statistical parameters including central tendency (e.g. means) or other basic estimates (e.g. regression coefficient) AND variation (e.g. standard deviation) or associated estimates of uncertainty (e.g. confidence intervals)
☐	☒	For null hypothesis testing, the test statistic (e.g. *F*, *t*, *r*) with confidence intervals, effect sizes, degrees of freedom and *P* value noted<br>*Give P values as exact values whenever suitable.*
☒	☐	For Bayesian analysis, information on the choice of priors and Markov chain Monte Carlo settings
☒	☐	For hierarchical and complex designs, identification of the appropriate level for tests and full reporting of outcomes
☐	☒	Estimates of effect sizes (e.g. Cohen's *d*, Pearson's *r*), indicating how they were calculated

Our web collection on statistics for biologists contains articles on many of the points above.

Software and code

Policy information about availability of computer code

Data collection	Sequencing reads were undertaken with the Hiseq-2500 sequencer (DAP-Seq and RNA-seq data); Bio-Rad CFX Manger v3.1 (qPCR data); Leica TCS SP5 (subcellular location);3000 Rice Genomes Project (genomic data of Rice 3k population);Rice Haplotype Map Project database (http://www.ncgr.ac.cn/RiceHap3).
Data analysis	Tassel 5 were used for GWAS. Microsoft Excel 2010, GraphPad Prism v8.0.1 and SPSS v17.0 (one-way ANOVA, Duncan's multiple range test) were used for statistical analysis. MEME chip and ChIPseeker were used for Motif prediction. 3DHISTECH and Caseviewer2.4 were used for FISH. R package pegas (version 4, paradis, 2010) was used for average nucleotide diversity and haplotype network of OsGATA8. MEGA7 (version 7.0) was used for constructing phylogenetic tree of OsAMTs.

For manuscripts utilizing custom algorithms or software that are central to the research but not yet described in published literature, software must be made available to editors and reviewers. We strongly encourage code deposition in a community repository (e.g. GitHub). See the Nature Portfolio guidelines for submitting code & software for further information.

Data

Policy information about availability of data

All manuscripts must include a data availability statement. This statement should provide the following information, where applicable:

- Accession codes, unique identifiers, or web links for publicly available datasets
- A description of any restrictions on data availability
- For clinical datasets or third party data, please ensure that the statement adheres to our policy

Data supporting the findings of this work are available within the paper and its Supplementary Information files. A reporting summary for this article is available as a Supplementary Information file. The datasets and genetic materials generated and analyzed during the current study are available from the corresponding author upon request. Sequence data of rice varieties can be found in our previous study (https://doi.org/10.1038/s41467-019-13187-1). Sequence data from this study can be found in the MSU database (http://rice.plantbiology.msu.edu/) under the following accession numbers: OsGATA8 (LOC_Os01g24070), OsTCP19 (LOC_Os06g12230), OsAMT3.2 (LOC_Os03g62200) and OsAMT1.2 (LOC_Os02g40730). Sequence for constructing the phylogenetic tree of AMTs (Extended Data Fig. 9) can be found in The Arabidopsis Information Resource database (https://www.arabidopsis.org/) or the MSU database (http://rice.plantbiology.msu.edu/). The DAP-sequencing and RNA-sequencing data have been uploaded to the website NCBI SRA (Sequence Read Archive) Database (https://www.ncbi.nlm.nih.gov/sra/PRJNA1103982 and https://www.ncbi.nlm.nih.gov/sra/PRJNA1104402). The source data are provided with this paper.

Research involving human participants, their data, or biological material

Policy information about studies with human participants or human data. See also policy information about sex, gender (identity/presentation), and sexual orientation and race, ethnicity and racism.

Reporting on sex and gender	No human participants involved.
Reporting on race, ethnicity, or other socially relevant groupings	No human participants involved.
Population characteristics	No human participants involved.
Recruitment	No human participants involved.
Ethics oversight	No human participants involved.

Note that full information on the approval of the study protocol must also be provided in the manuscript.

Field-specific reporting

Please select the one below that is the best fit for your research. If you are not sure, read the appropriate sections before making your selection.

☒ Life sciences ☐ Behavioural & social sciences ☐ Ecological, evolutionary & environmental sciences

For a reference copy of the document with all sections, see nature.com/documents/nr-reporting-summary-flat.pdf

Life sciences study design

All studies must disclose on these points even when the disclosure is negative.

Sample size	We planted 117 rice varieties and transgenic rice lines in the high nitrogen (HN, 300 kg/ha nitrogen fertilizer) and low nitrogen (LN, 0 kg/ha nitrogen fertilizer) fields. Samples sizes for each experiment is indicated in legends. Each experiment contains at least three biololgical replicates.
Data exclusions	No data was excluded from the analyses.
Replication	All experiments in this study were repeated independently at least three times. For qRT-PCR at least three biologically independent samples were used each time. For subcellular location, field and physiological and biochemical experiments, the results representative of three independent experiments. The number of biological replicates in each experiment was indicated in the legends.
Randomization	For field experiments, the varieties and transgenic lines were grown in a completely-randomized block design with four replicates.
Blinding	The investigators were blinded to group allocation during data collection.

Reporting for specific materials, systems and methods

We require information from authors about some types of materials, experimental systems and methods used in many studies. Here, indicate whether each material, system or method listed is relevant to your study. If you are not sure if a list item applies to your research, read the appropriate section before selecting a response.

Materials & experimental systems

n/a	Involved in the study
☐	☒ Antibodies
☒	☐ Eukaryotic cell lines
☒	☐ Palaeontology and archaeology
☒	☐ Animals and other organisms
☒	☐ Clinical data
☒	☐ Dual use research of concern
☐	☒ Plants

Methods

n/a	Involved in the study
☒	☐ ChIP-seq
☒	☐ Flow cytometry
☒	☐ MRI-based neuroimaging

Antibodies

Antibodies used: DYKDDDDK Tag Antibody (MA1-91878-BTIN) was used for IP.

Validation: DYKDDDDK Tag Antibody (MA1-91878-BTIN) can be found at ThermoFisher (https://www.thermofisher.cn/)

Dual use research of concern

Policy information about dual use research of concern

Hazards

Could the accidental, deliberate or reckless misuse of agents or technologies generated in the work, or the application of information presented in the manuscript, pose a threat to:

No	Yes
☒	☐ Public health
☒	☐ National security
☒	☐ Crops and/or livestock
☒	☐ Ecosystems
☒	☐ Any other significant area

Experiments of concern

Does the work involve any of these experiments of concern:

No	Yes
☒	☐ Demonstrate how to render a vaccine ineffective
☒	☐ Confer resistance to therapeutically useful antibiotics or antiviral agents
☒	☐ Enhance the virulence of a pathogen or render a nonpathogen virulent
☒	☐ Increase transmissibility of a pathogen
☒	☐ Alter the host range of a pathogen
☒	☐ Enable evasion of diagnostic/detection modalities
☒	☐ Enable the weaponization of a biological agent or toxin
☒	☐ Any other potentially harmful combination of experiments and agents

Plants

Seed stocks	The seeds of the 117 accessions were collected, stored, and supplied by the State Key Laboratory of Crop Genetics & Germplasm Enhancement and Utilization, Jiangsu Collaborative Innovation Center for Modern Crop Production, Nanjing Agricultural University, China.
Novel plant genotypes	We used the CRISPR/Cas9 technology in generating genome-edited plants. Agrobacterium-based transformation was used to generate the overexpression lines and the genome-edited rice plants.
Authentication	PCR and Sanger sequencing was used to verify the genome edits generated at the designated genomic targets. RT-qPCR are used to verify the expression of the transgenes.
