# Supplementary figures and images for: The elite haplotype OsGATA8-H coordinates nitrogen uptake and productive tiller formation in rice
Source: Nat Genet. 2024 Jun 13;56(7):1516–26. doi: 10.1038/s41588-024-01795-7 (PMC11250373; doi:10.1038/s41588-024-01795-7)

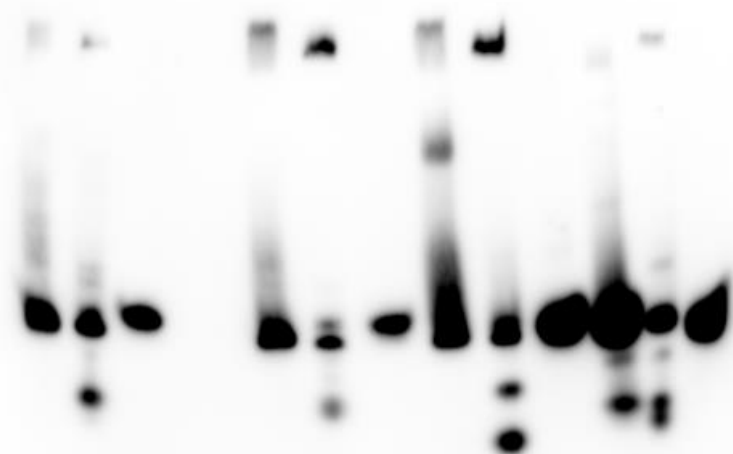

Supplement: Supplementary file 23 — Unprocessed EMSA blots for Fig. 2d. [file 41588_2024_1795_MOESM23_ESM.pdf]

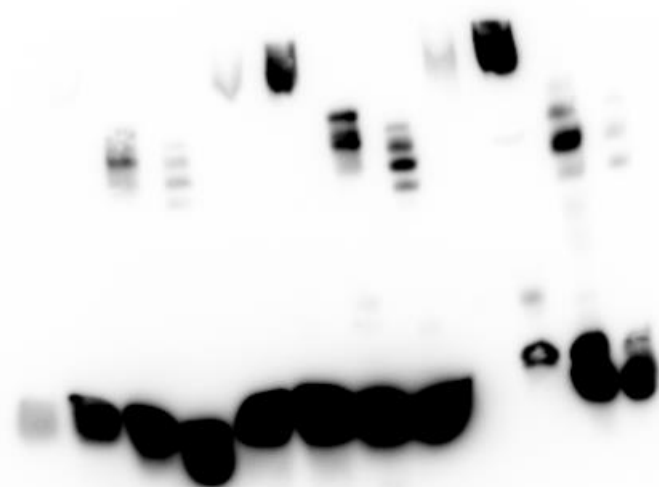

Supplement: Supplementary file 24 — Unprocessed EMSA blots for Fig. 3b. [file 41588_2024_1795_MOESM24_ESM.pdf]
